# Notes and News

**Published:** 1905-02-18

**Authors:** 


### Notes and News.

A vacancy is advertised for a medical superintendent for the Inebriates' Reformatory, Cattal, near York. Applicants must be fully qualified and registered, and the salary is £250 per annum, with residence, board, and all attendant expenses.

As we go to press we are informed that the matron of the Queen's Jubilee Hospital, and the whole of the medical staff, with the exception of Mr. Fitzroy Benham and the dentist, have resigned. No particulars are given, but the reasons for these resignations may have an important bearing upon the future of this institution, the administration of which has given rise to much criticism in the past.

The death of Mr. Luther Holden will cause regret to a very large professional circle amongst whom he has been an interesting personality for many years. Mr. Holden was born in 1815 and practically during the whole of his medical career he has been associated with St. Bartholomew's Hospital, though intervals occurred when he studied in Edinburgh, France, and Germany. He became F.R.C.S. as long ago as 1844, and was president in 1879. He was Senior Consulting Surgeon at St. Bartholomew's Hospital at the time of his death. He was the author of several well-known medical works.

The subject of the feeding of school children occupied some of the time and interest of the Conference on School Hygiene, which recently met in London, and was well presented in a carefully reasoned paper by Dr. A. K. Chalmers, the medical officer for health of Glasgow. In this it was shown that the physical development and nutrition of school children are related to an economic standard of the family life, which may readily be expressed, and that this standard is, in many instances, of so low a level that the mental and physical condition of the children renders them incapable of utilising their educational opportunities. This means, first, the non-success of much educational energy, as is shown by the small proportion of underfed children who reach a reasonable standard of proficiency; and, secondly, the development of a class of industrial inefficients, from whom the vicious cycle is again commenced. Dr. Chalmers concludes that to prevent these results a supply of food, and, by implication, the organised feeding of school children, in certain districts, is essential, and that, with a view to ascertain the distribution of underfed children, a systematic inspection of the schools is necessary.

The cases of typhoid at Lincoln number upwards of 600. Fortunately the type is a mild one, and the percentage of fatal cases small. The Government experts have not yet reported officially on the water, which is regarded in the meanwhile with suspicion, and avoided as far as possible.

We regret to record the death of Dr. Robson Roose, at the comparatively early age of 59. Dr. Robson Roose received his medical education at Guy's, Paris, and Brussels. He held the M.D. degree of the latter university. He was F.R.C.S. England, and M.R.C.P. Edinburgh. He was a popular physician in society, and devoted much attention to the conditions affecting the daily life of Londoners.

The late Mr. J. H. Lucking, of Streatham Hill, has bequeathed £2,000 each to the Asylum for Idiots, Earlswood, the National Society for the Employment of Epileptics, the British Home for Incurables, St. George's Hospital, King's College Hospital, the London, St. Mary's, and the Middlesex Hospitals, the Brompton Hospital for Consumption, the National Hospital for the Paralysed and Epileptic, and the Royal Hospital for Incurables, Putney. He bequeathed £1,000 each to the Charing Cross, Guy's, the Metropolitan, the London Free, the Seaman's, Westminster, University College, East London, Evelina, Victoria, Samaritan Free, Cancer, Royal London Ophthalmic, Royal Sea Bathing, and the Female Lock Hospitals, the Hospital for Sick Children, the Hospital for Women, the City of London Hospital for Diseases of the Chest, the All Saints' Convalescent Home, Eastbourne, and the Bexhill Convalescent Home. To several other medical charities he left £500.

A letter signed by a Fellow of the Royal Sanitary Institute, which appeared in the *Times* of February 11th, brings into prominent notice an example of defective sanitary arrangements in a lodging-house at one of our Universities. The bad sanitary condition recorded is serious enough in itself, but the ineffective methods of the health authorities to protect the public which is revealed is still more so. Apparently a medical officer of health may inspect the arrangements in a dwelling, but if an inquiry is made by a would-be inhabitant of the dwelling the medical officer is justified in refusing information. The question is one of vital interest to all those who hire apartments. How many cases of illness might not be traced to sojourns in unhealthy houses it would be difficult to estimate, nevertheless the public is singularly apathetic in the matter. Some of the better class owners of apartment-houses do offer to produce a satisfactory sanitary certificate, but their number is very small, and at a crowded seaside resort the meek paterfamilias would not dare to impose a condition upon an imposing landlady who condescends to provide him with accommodation for which he knows the demand is greater than the supply. The Royal Sanitary Institute might render a service to the country by securing legislature which would require that all who receive lodgers should be required to show a sanitary certificate on demand. The responsibility attaching to the signature of such a document might be very stimulating and healthy.

